# Supplementary material for: Appendiceal Intussusception: A Rare Diagnosis and the Role of Imaging in Its Detection
Source: Diagnostics (Basel). 2025 Jan 23;15(3):256. doi: 10.3390/diagnostics15030256 (PMC11816855; doi:10.3390/diagnostics15030256)
Supplement: Supplementary file 1 [file diagnostics-15-00256-s001.zip › diagnostics-3426101-supplementary.pdf]

**Type I**	The tip of the appendix invaginates into the proximal part	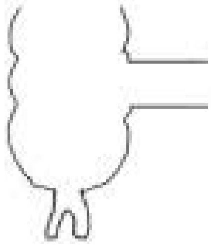
**Type II**	Invagination occurs along the longitudinal axis.	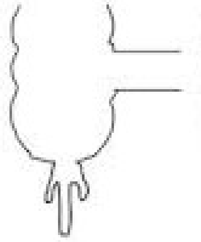
**Type III**	Invagination starts at the junction of the appendix and cecum.	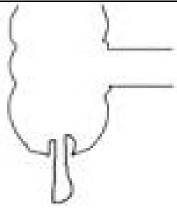
**Type IV**	Retrograde intussusception, where the proximal part invaginates into the distal part.	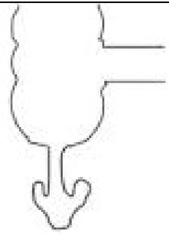
**Type V**	Complete invagination of the appendix into the cecum.	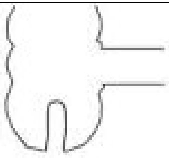

Table S1. Schematic presentation of appendiceal intussusception
